# Application of deep metric learning to molecular graph similarity

**DOI:** 10.1186/s13321-022-00595-7

**Published:** 2022-03-12

**Authors:** Damien E. Coupry, Peter Pogány

**Affiliations:** Data and Computational Sciences, GlaxoSmithKline, Stevenage, UK

**Keywords:** Metric learning, Similarity, Graph neural networks, Deep learning

## Abstract

Graph based methods are increasingly important in chemistry and drug discovery, with applications ranging from QSAR to molecular generation. Combining graph neural networks and deep metric learning concepts, we expose a framework for quantifying molecular graph similarity based on distance between learned embeddings separate from any endpoint. Using a minimal definition of similarity, and data from the ZINC database of public compounds, this work demonstrate the properties of the embedding and its suitability for a range of applications, among them a novel reconstruction loss method for training deep molecular auto-encoders. Finally, we compare the applications of the embedding to standard practices, with a focus on known failure points and edge cases; concluding that our approach can be used in conjunction to existing methods.

## Introduction

Quantifying the similarity of chemical structures has been frequently used in drug discovery for decades [55], and has often been adopted as a design principle for lead optimization [29, 34] under the assumption that similar molecules have a higher probability of exhibiting similar properties than dissimilar ones [27, 36, 40]. Indeed, the successful use of bioisosterism in drug development makes heavy use of the concept [32, 39], to the point that similarity is sometimes defined as a consequence of the properties, rather than the cause [5]. Most of the benchmarks for chemical structure similarity rely on this definition to compare methods [25, 44, 46], driven in part by the availability of public activity datasets [20]. Yet, pitfalls such as so-called “activity cliffs” [35, 49, 50] should moderate the confidence in the underlying principle. Furthermore, other use cases of similarity exist, and are not captured by the similar properties paradigm: patent mining and infringement prediction [43], building block selection for synthesis, retrosynthesis and scaffold hopping [8, 9, 14], molecular generation evaluation [37], etc. A “good” measure of similarity should ideally show equal performance in all these applications, never relying too much on any one definition or type of benchmark. On the practical side, similarity can be more generally understood as the combination of a molecular representation and an appropriate metric [34]. Today, the combination of two-dimensional molecular circular fingerprints [13, 45] with the Tanimoto coefficient [3] is still the most widely used, and generally hard to outperform in traditional benchmarks [42]. However, these methods suffer from a number of identified drawbacks, regularly analysed but difficult to route around in the absence of a more general representation [16, 18]. Most of the recent efforts to develop original molecular encodings focus on the relational nature of molecules as seen in a 2D context. By considering structures as a graph with atoms as nodes and bonds as edges, we can draw on the considerable field of extant work on graph similarity in general: computationally expensive graph edit distance, graph isomorphism quantification or maximum common subgraph [6, 11, 12, 15, 19], graph kernels for similarity [28], and the increasingly popular deep learning algorithms [33]. The latter rely on embeddings learned from variational reconstruction tasks [26], end-to-end property predictions [10]. In this work, we leverage the ability of graph neural networks from the Deep Graph Library [31, 53] to learn chemical structures embeddings using the triplet loss [47], to our knowledge the first such use of it. This is an application of the deep metric learning approach, a popular architecture from facial recognition [2]; where a feature space is conditioned with the euclidean distance, making it a metric space suitable for similarity quantification. A training dataset is constructed automatically using a minimal definition of molecular similarity and public compounds. We show that these embeddings satisfy the conditions to be considered an appropriate encoding of molecular graph similarity information, applicable in both traditional benchmarks and novel applications.

**Fig. 1:**
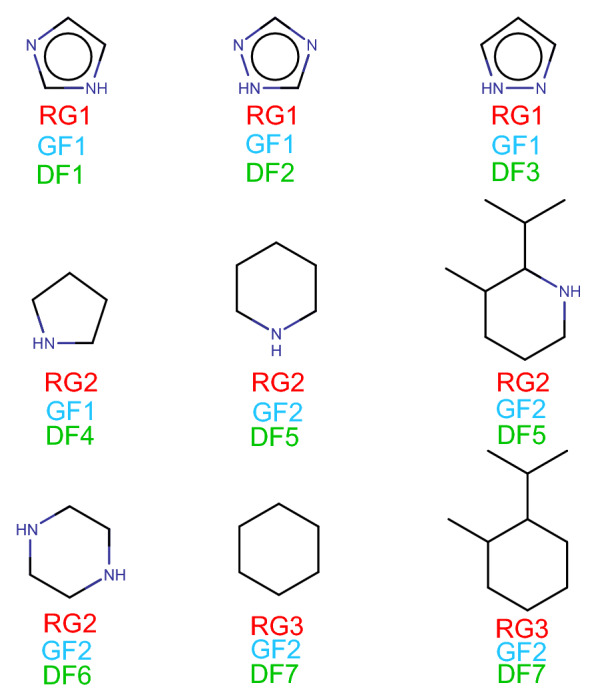
A comparison of the Reduced Graph (RG), Bemis-Murcko graph (GF) and detailed frames (DF) clusters. The numbers after the character show the cluster. RG1 is a cluster of aromatic ring containing compound which contain hydrogen bond donor and acceptor. RG2 are aliphatic rings with hydrogen bond donors, RG3 are aliphatic rings without feature. There are only two graph frame clusters: 5-membered rings (GF1) and 6-membered rings (GF2). Detailed frames are only identical, if the compounds differ in ring substituents connected to rings with single bonds (DF5 and DF7)

**Fig. 2:**
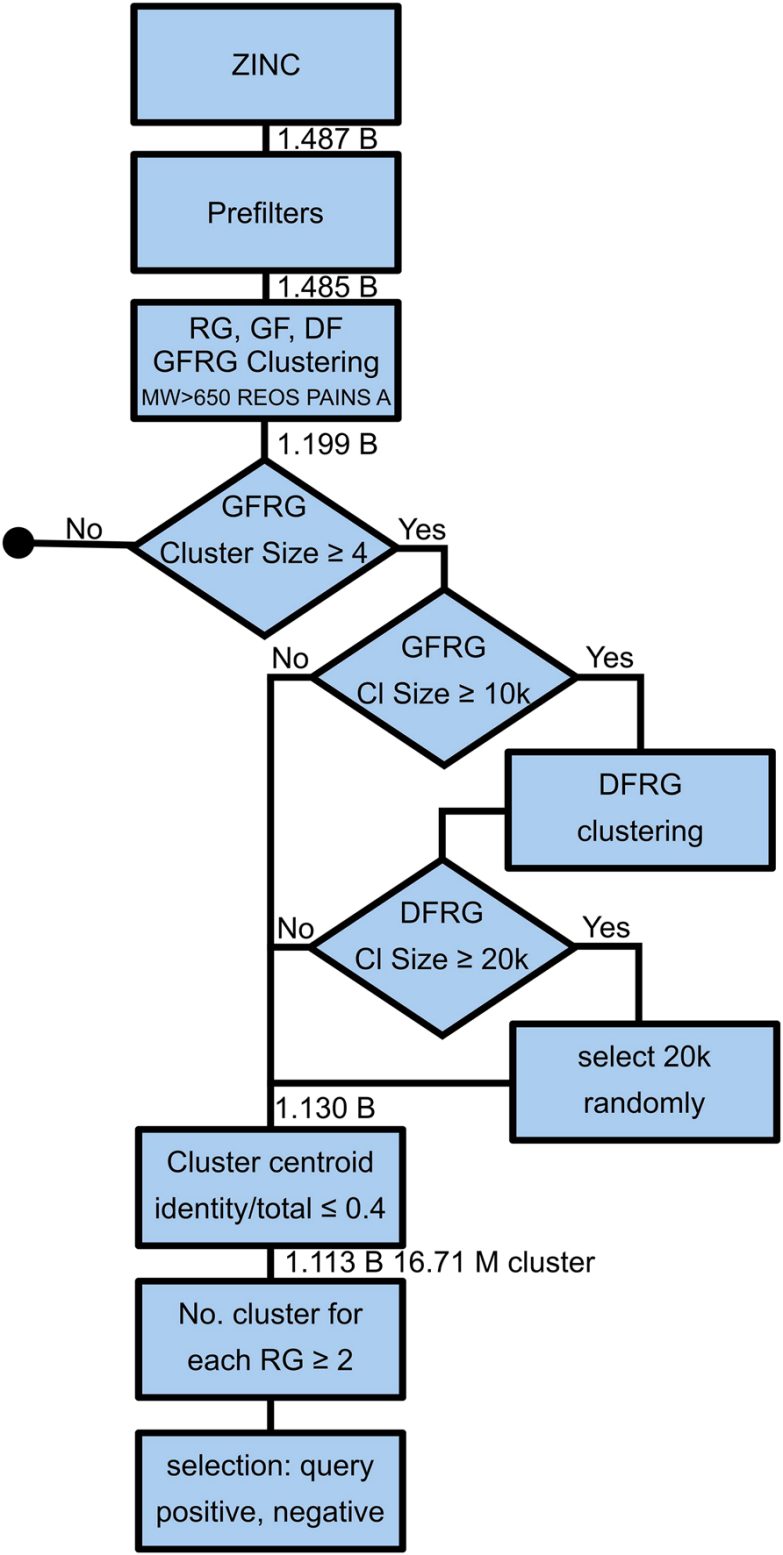
The process diagram of data preparation

**Fig. 3:**
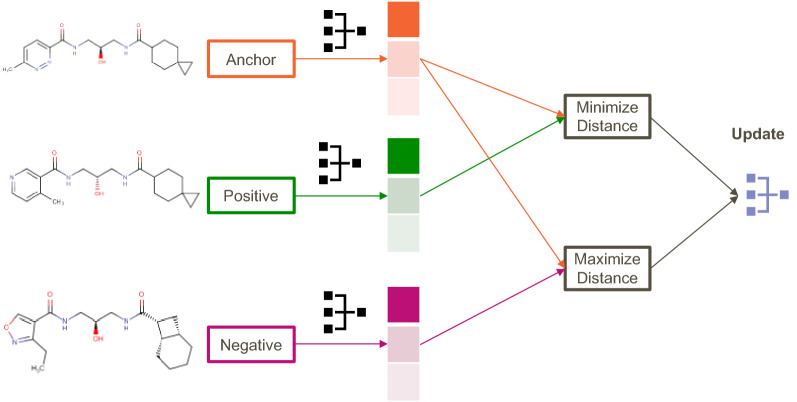
The architecture of the triplet loss embedding during training

## Experiments

### Dataset generation

The ZINC database was downloaded (1.487 billion compounds) [48] and processed as follows. Parent structures were created, bad valencies, compounds with poorly defined bonds, isotope labelled compounds and compounds containing elements other than N, O, C, S, F, Cl, Br and I were removed. This initial filtering removed around 2 million compounds. Reduced Graphs [21, 24], Bemis-Murcko graph and Bemis-Murcko detailed frames [4] were generated for each compound. In the Reduced Graph, the full molecular graph is reduced to pharmacophore feature type nodes. The Bemis-Murcko graph frame contains the anonymous frame of the molecule without the side chains, atom types and bond orders, whereas the Bemis-Murcko detailed frame contains the frame of the molecule (side chains removed) with atom types and bonds marked. Comparison of these molecular representations is given on Fig. 1.

REOS [52] and PAINS A [1] filters were applied on the remaining compounds and molecular weight (MW) was calculated to remove everything with MW>650 daltons, thus keeping 1.199 B compounds. Compounds were clustered in three ways:

1. Having the same Reduced Graph and Graph Frame (GFRG)
2. Having the same Reduced Graph and Detailed Frame (DFRG)
3. Having the same Reduced Graph (RG)

Most of the processing after this was done using BIOVIA Pipeline Pilot [7]. All compounds belonging to a GFRG cluster with less than 4 members were removed. In the case of compounds belonging to GFRG clusters with more than 10k members, DFRG clusters were used in place of GFRG. For DFRG clusters, a maximum size of 20k members was established, with random subsampling performed on clusters above this limit. 1.13 billion compounds remained and cluster centeroids were assigned to them. Cluster Molecules component of BIOVIA Pipeline Pilot [7] was used to determine the cluster centroids for each cluster defined above (ECFP4 and heavy atom count was used for getting the centroids). For every cluster the number of identities was calculated. If the number of identities was larger than 0.4, all the cluster elements were discarded. 1.113 billion compounds remained in 16.71 million clusters. The number of clusters for each Reduced Graph was calculated and only Reduced Graphs which have at least 2 clusters were kept (1.059 billion compounds).

The triplet loss trains networks by contrasting a reference structure with two additional compounds, called positive and negative controls. The positive control should be qualitatively similar to the reference. For this purpose, the two (reference and positive control) were selected randomly from within the same cluster (GFRG cluster for the initial smaller clusters, for the larger clusters, where GFRF cluster size $\ge$ 10,000, DFRG clusters are used). The negative control should, conversely, be less similar to the reference than the positive. Selecting a very different compound is not optimal, since the chemical space size increases towards larger dissimilarities. Thus, while it would be correct to choose a negative control from a different cluster, choosing a compound that has *some* similar features to the reference is more valuable for the training process and it is also more challenging for the training. Therefore we have randomly selected the negative control from a different cluster than the cluster of the reference, but their Reduced Graph should be the same. This way 12,361,633 triplets were created. A detailed schema of the data preparation can be seen on Fig. 2.

### Model training

For all training and benchmarking purposes, the random seed is fixed at 42 for repeatability, and the hyperparameters have been kept unoptimized and to the default values to prevent bias. To keep computation times short, only a random sample of 10% of triplets generated is used during training, the rest being kept for testing purposes. We used the DGL-Lifesci open source framework for computations on graphs, and its message passing neural network implementation (MPNNPredictor) [22] as model architecture. This type of model repeatedly accumulates bond information as well as node information based on connectivity, and has been used with great effect in state of the art QSAR applications [56]. We chose to use the default parameters and an output size equal to 16 as an embedding dimension (*n_tasks*). The input for such a model are molecular graphs, which are obtained using the CanonicalAtomFeaturizer and CanonicalBondFeaturizer from DGL. The details of what is included in the graphs features can be found in the DGL-lifesci documentation. These representations are regularized with a node ablation probability of 1% and edge ablation probability of 5%. At each step of the training, an instance of the MPNN is used to embed each of the three graphs of the input (anchor, positive and negative); the triplet margin loss from pytorch [38] then updates the weights of the network to maximize the distance between the anchor and negative, while minimizing the distance between the anchor and the positive, as seen in Fig. 3.

The training used the pytorch-lightning framework [17] with a 25 epochs early stopping criterion, the Adam optimizer with the default learning rate of $10.0^{-3}$, and took two days on an Nvidia GEFORCE1080 GPU with a batch size of 128. For more details, hyperparameters, and training curves, please refer to the project’s github page (https://github.com/DCoupry/ChemDist_paper).

### Benchmarks choice

The benchmarks for the present use case should optimally measure a number of things:

- The performance on popular applications; here the activity classification tasks such as the ones described in Riniker et al.[44].
- The performance on edge cases, such as the ones described in Flower et al. [18], particularly when the failure of traditional fingerprint based similarity measure is due to the basic technique of fragmentation.
- The condition of graph isomorphism: the ordering of the molecule atoms and bonds should have no influence on the embedding.

Additionally, *desired* properties of an encoding come from the coupling with a metric. In particular, using a euclidean distance metric on a well defined euclidean vector space gives rise to a number of interesting properties:

- very fast querying and operations
- Similarity can be defined with respect to geometric elements: around a barycentre, along a path between molecules, within a cone, etc.
- the space and metric together are unbound in value for dissimilarity: there are many more ways of being dissimilar than similar, and the distances distribution could reflect that.

## Results

### Activity prediction tasks benchmarking

While an imperfect measure of fitness for any new chemical embedding, the dominance of benchmarking platforms making use of a variety of activity prediction datasets makes it an obligatory step in evaluating any new contribution. In particular, it enables two separate conclusions to be reached:

1. Whether the information contained in the embedding is sufficient to fit models successfully, regardless of compared performance
2. Whether these models are statistically different from references to demonstrate the originality of the embedding

**Fig. 4:**
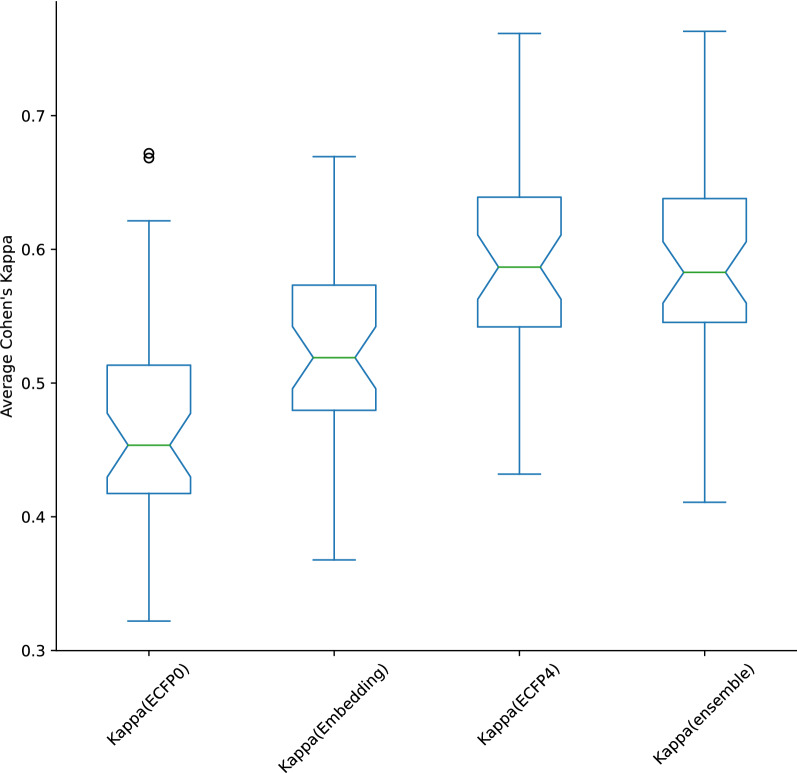
Performance in activity classification tasks from ChEMBL28

**Fig. 5:**
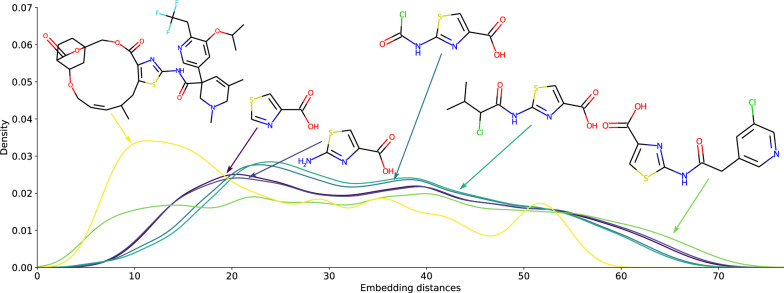
Distribution of embedding distances of 5 references compounds to a diverse set of 120k compounds from the Zinc database

**Fig. 6:**
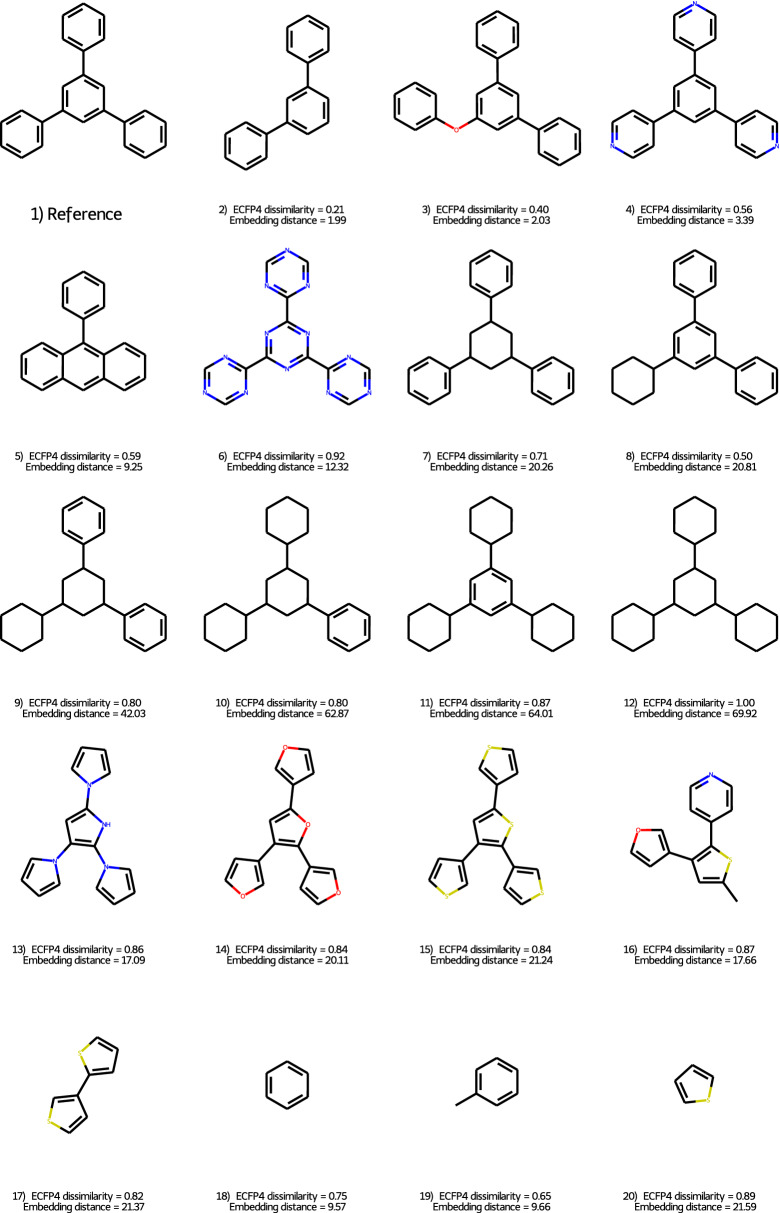
Selection of pairwise comparisons illustrating a diverse set of molecular similarities

**Fig. 7:**
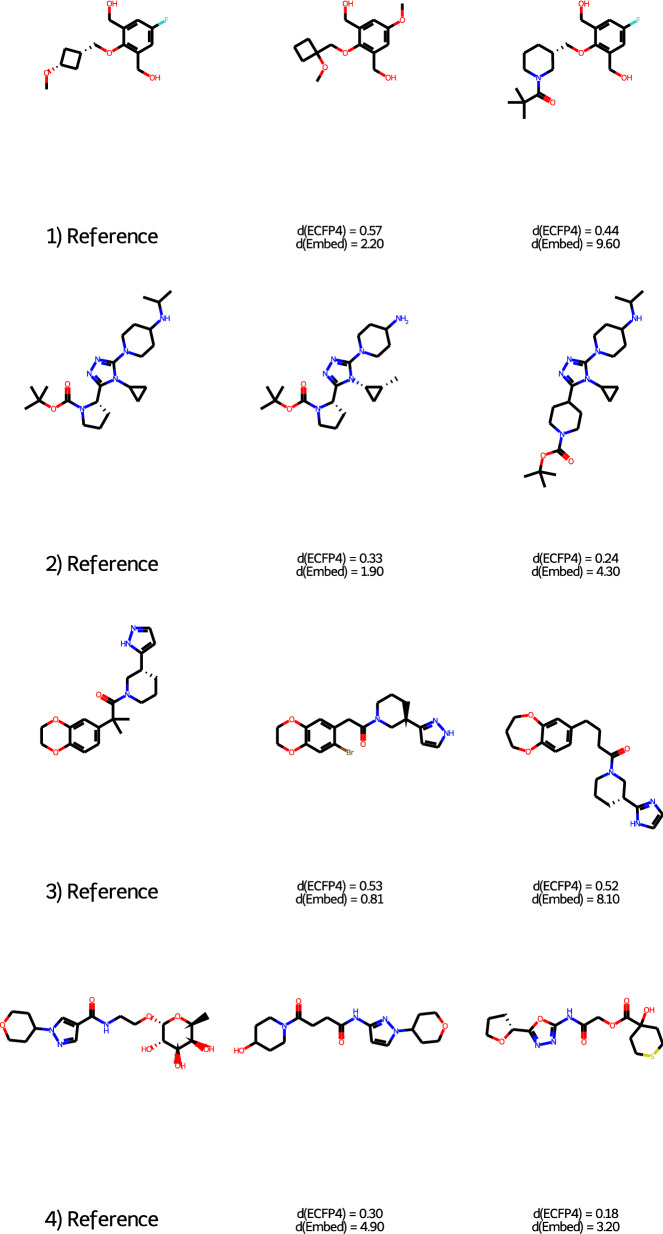
Selection of triplets not seen during the training where ECFP4 and the triplet embedding does not agree in the order of the positive (structure in the middle for each row) and negative (right hand side structure) controls

**Fig. 8:**
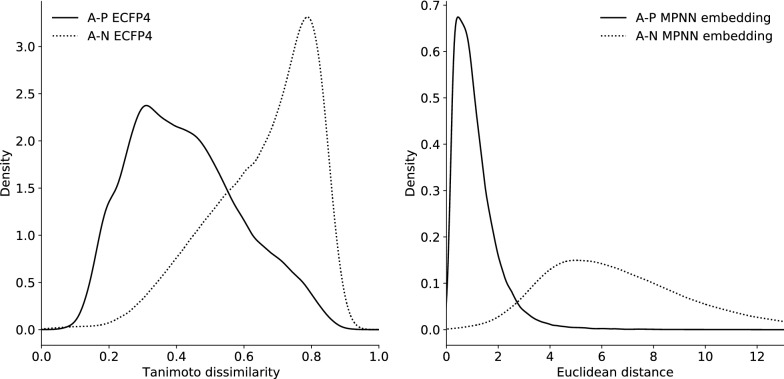
Comparison of the similarity distributions on unseen triplets

**Fig. 9:**
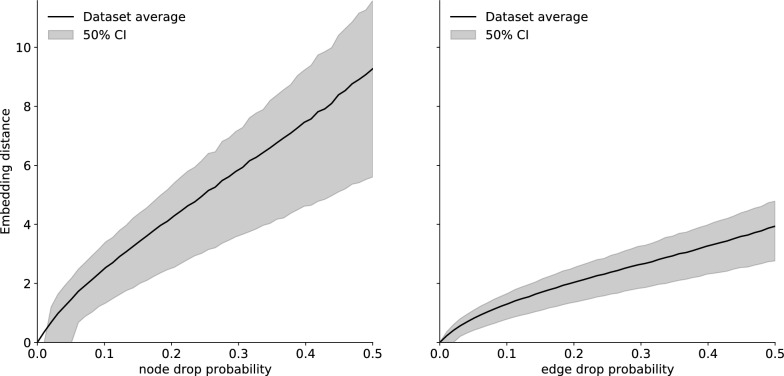
Effect of random element deletion on embedding distance. No comparison with ECFP4 could be obtained due to the overwhelming rate of invalidity of the resulting structures

To answer the second query, it is necessary to benchmark models on a suitably high number of instances for each class. For this purpose, a dataset of IC50 activities was extracted from the ChEMBL28 database. All targets with a unique structure count between 5k and 20k were kept, with activity threshold automatically set at the 75th percentile of the PIC50 values if and only if this is superior by at least one standard deviation from the minimum value and maximum value. This classification task was modelled by a k-nearest neighbours classifier from the scikit-learn python package [41], trained on ECFP0 and ECFP4 fingerprints from the rdkit package [30], as well as on learned embeddings. Only targets with an ECFP0 5-fold stratified cross validation Cohen’s Kappa score above 0.25 were kept, to constrain the benchmark tasks to be relatively hard but tractable, resulting in a set of 55 targets. For each triplet of models, the Cochran’s Q test was applied to verify statistical difference. The p-values of 30 tested targets were <0.05 and sufficient to reject the null hypothesis that all the models were equivalent. Subsequent confirmation with pairwise McNemar tests with Bonferroni correction show the embedding models to be the source of the statistical difference, thus answering our second point. The performances on this final set of 30 targets are shown in Fig. 4: while ranking below ECFP4 in average, the embedding systematically outperforms ECFP0, confirming that the information extracted from the graphs can be used by subsequent algorithms. This answers our first point to our satisfaction. Furthermore, a simple ensembling of models built on ECFP4 and the embedding results in a modest improvement in 15 models, showcasing the potential benefits of integrating our method to existing workflows.

### Failure points of circular fingerprints

One noted effect of the bit-string fingerprints is the skewing effect of size on the distribution of similarities as illustrated in Fig. 6 of Flowers et al. [18]. Applying the same reference set of compounds for comparison on a diverse set of molecules using the MPNN learned embedding leads to a much better shape of the distributions. While the larger molecule has a more chaotic profile of similarity (probably due to the fact that the larger a structure, the more ways for something to be similar to it), it otherwise seems independent from the size of the molecules. This is shown in Fig. 5.

Another point where fingerprints fail to accurately describe molecular similarity is the case of molecules with repeated motifs. When using Tanimoto similarity of circular fingerprints in bit string form, the similarity tapers off quickly to a fixed non-zero value. The learned embedding is immune to this effect. Likewise, the insertion of moieties within a scaffold has an unduly small effect when it does not perturb the fragmentation of the structure by fingerprints, but is correctly shown to matter a lot by the embedding. In addition, it also retains the concepts of fragments, aromaticity, and some level of isosterism. Some examples illustrating these points are shown in Fig. 6.

The usual dissimilarity cutoff values in case of ECFP4 fingerprints are between 0.2-0.4 (anything below this is considered to be similar). At these low values (structures 2 and 3 on Fig. 6) the triplet embedding distance agrees well with ECFP4 dissimilarity. Structures 6, 9-12, 17 and 20 are largely dissimilar according to ECFP4, having a dissimilarity at least or above 0.8. As we can see the triplet embedding distance discriminates between these structures much more than ECFP4. It prefers generally the aromatic structures with similar arrangements against the aliphatic rings, what is expected from the nature of reduced graphs. The 5-membered aromatic rings (e.g. structure 13-16) are closer based on the triplet embedding to the original Reference than the similarly arranged structures with at least 2 aliphatic rings (structures 9-12). This is not so clear in case of ECFP4, which does not distinguish between structures 9 and 10 (both having and ECFP4 dissimilarity of 0.80, whereas the triplet embedding clearly showing that more aliphatic rings are less similar 42.03 vs. 62.87 for 9 and 10, respectively) and creates a large difference between similar structures 7 and 8 (0.71 vs. 0.50 for ECFP4). The 2nd most dissimilar structure based on ECFP4 is structure 6 (with a large dissimilarity of 0.92), whereas the triplet embedding shows a not too large dissimilarity (12.32). This later is not surprising, although the arrangement of the ring systems is the same and the molecular shape is similar, the non-featurized ECFP4 only understands that the rings changed completely between the reference and structure 6 and it does not find a lot of similarity between the benzene and triazine rings.

To show further differences between the ECFP4 and the triplet embedding a randomly selected set of 100,000 triplets unused in the training process was utilized to calculate both the ECFP4 dissimilarities and the triplet embedding distances for the positive and negative controls in respect to the reference (anchor). The experiment showed that both ECFP4 dissimilarity and the triplet embedding determined the correct order (positive control has lower distance than the negative) for 89,133 triplets, showing that in most cases both work fine. Not surprisingly, ECFP4 failed more often (9911 cases), whereas the triplet embedding failed only for 956 cases. There are 428 cases were both failed. Although this is not a quantitative performance investigation for the two distance metrics, it can give us insight about their weak points. In Fig. 7 we show 4 examples (the whole list is in the github repository) where one of the descriptors failed to give the correct order. In case of triplet 1), ECFP4 predicted that the negative control (right hand side) is closer to the reference than the positive one (middle). Since in case of the negative control the left hand side of the molecule changes (4-membered ring is changed to a 6-membered ring), for a chemical series point of view this change is larger than the changes in the side chains, which can be seen in case of the positive control. Triplet 2) shows a similar case, where ECFP4 fails to properly give the order. Here the failure is caused both by feature repetition and a relatively small change of the ring size. In case of the negative control, the piperidine ring appears two times in the molecule. The ECFP4 used here (and in many virtual screening and similarity searching experiments) does not contain feature counts, therefore the sensitivity to feature repetition is low (see Fig. 2 in reference [18]). Triplet 3) shows also an example where the ring size changes, but here the ECFP4 dissimilarities are almost identical, although there are not only changes in the side chains, but a linker extension, a ring extension and pyrazole ring is changed to an imidazole ring in case of the negative control. A different case is triplet 4), where the triplet embedding failed to properly determine the order. As it can be seen, both negative and positive controls have larger changes, although the two rings on the right hand side are the same for the positive control, their connection is different. The amide bond is reversed in both the positive and negative controls compared with the reference structure, the linker has the same length, but different groups and the left hand side ring system is largely different for both the positive and negative controls. Both ECFP4 and the triplet embedding gave a larger distance for these two structures. The insensitivity on the orientation of the amide groups is a well known issue of the reduced graphs. Triplet 4) can be considered as a bad example, since both for positive and negative control there are large changes in the core of the structure. Large part of those structures where the triplet embedding failed are similar to this, i.e. the positive controls and the negative controls are both in not too close distance to the reference and in some cases they are more similar to each other than to the reference. Preparing better the training set might solve part of the issues, but a small number of “wrong” examples might always get into the data set.

### Additional properties

As stipulated earlier, the distribution of similarities should be notably different between positive examples and negative examples: the first distribution should show a sharp peak around optimal similarity, and the second should display a long tail representing the many different sources of dissimilarity. After applying both the ECFP4 Tanimoto dissimilarity coefficient comparison and the learned MPNN (triplet) embedding to unseen triplets of our generated dataset, we indeed see such a behaviour illustrated in Fig. 8.

Another critical desired property for a novel molecular distance measure is the ability to correctly compare partial and *chemically invalid* molecular graphs and provide gradient information. This leads to the important fact that trained embeddings are essentially derivable reconstruction loss with a quadratic energy surface, with widespread potential applications. For example:

- Accelerated training of reconstruction based molecular generators such as variational auto-encoders.
- Additional information in tasks such as missing edge and node prediction.
- Chemical subspace constraints for conditional molecular generators

These tasks are deeply unsuitable to traditional fingerprints or property based similarity : for most of the training process, the molecular graphs on which computation happens are completely invalid, the chemical information on what is a molecule still being accrued. Yet a learned embedding, as is shown in Fig. 9, is very robust to node and edge deletion, demonstrating a quasi linear distance relationship with the number of deleted elements. This is an exciting property, and we look forward to seeing it explored further.

Finally, a critical property of the embedding is its ability to be used in conjunction with transfer learning [51, 54], and be retrained on particular subsets of the chemical space according to tailored similarities obtained from SAR, Molecular Matched Pairs [23], or a more complex multiple-parameters function. Such a retrained model would retain the general concepts of molecular graph similarity while quickly converging to a more appropriate representation of the problem at hand, thus sparing resources in training and data gathering.

## Conclusions

We have shown that using the triplet margin loss jointly with molecular graph based deep neural networks trains latent representations that satisfy the many definitions of chemical similarity. A naive example of such an embedding was trained with no hyperparameters optimization on a dataset constructed from public molecules and some basic concepts of graph similarity. This naive example compares acceptably out of the box with the accepted standard of circular fingerprints Tanimoto scores, while possessing many additional properties such as being derivable or retrainable. We believe such properties may be of great use to train reconstruction based molecular generators.

## Data Availability

All code and data is available on https://github.com/DCoupry/ChemDist_paper under an Apache 2 license (GlaxoSmithKline copyright) and is sufficient to reproduce our conclusions and graphs.

## Abbreviations

QSAR: Quantitative Structure Activity Relationship
GNN: Graph Neural Network
MPNN: Message Passing Neural Network
RG: Reduced Graph
DF: Detailed Frame
GF: Bemis Murcko Graph
ECFP: Extended Connectivity Fingerprint

## References

[1] Baell JB, Holloway GA (2010). New substructure filters for removal of pan assay interference compounds (pains) from screening libraries and for their exclusion in bioassays. J Med Chem.

[2] Bai Y, Ding H, Bian S, Chen T, Sun Y, Wang W (2019) Simgnn: A neural network approach to fast graph similarity computation. In: Proceedings of the Twelfth ACM International Conference on Web Search and Data Mining, pp 384–392

[3] Bajusz D, Rácz A, Héberger K (2015). Why is tanimoto index an appropriate choice for fingerprint-based similarity calculations?. J Cheminformatics.

[4] Bemis GW, Murcko MA (1996). The properties of known drugs. 1. Molecular frameworks. J Med Chem.

[5] Bender A, Glen RC (2004). Molecular similarity: a key technique in molecular informatics. Org Biomol Chem.

[6] Berretti S, Del Bimbo A, Vicario E (2001). Efficient matching and indexing of graph models in content-based retrieval. IEEE Trans Pattern Anal Mach Intell.

[7] BIOVIA DS (2020). Discovery studio visualizer, release 2020.

[8] Boehm M, Wu TY, Claussen H, Lemmen C (2008). Similarity searching and scaffold hopping in synthetically accessible combinatorial chemistry spaces. J Med Chem.

[9] Böhm HJ, Flohr A, Stahl M (2004). Scaffold hopping. Drug Discov Today Technol.

[10] Brown N (2009). Chemoinformatics-an introduction for computer scientists. ACM Comput Surv.

[11] Bunke H, Allermann G (1983). Inexact graph matching for structural pattern recognition. Pattern Recognit Lett.

[12] Bunke H, Shearer K (1998). A graph distance metric based on the maximal common subgraph. Pattern Recognit Lett.

[13] Cereto-Massagué A, Ojeda MJ, Valls C, Mulero M, Garcia-Vallvé S, Pujadas G (2015). Molecular fingerprint similarity search in virtual screening. Methods.

[14] Coley CW, Rogers L, Green WH, Jensen KF (2017). Computer-assisted retrosynthesis based on molecular similarity. ACS Cent Sci.

[15] Dijkman R, Dumas M, García-Bañuelos L (2009) Graph matching algorithms for business process model similarity search. In: International conference on business process management, Springer, pp 48–63

[16] Dixon SL, Koehler RT (1999). The hidden component of size in two-dimensional fragment descriptors: side effects on sampling in bioactive libraries. J Med Chem.

[17] Falcon ea WA (2019) Pytorch lightning. GitHub Note. https://githubcom/PyTorchLightning/pytorch-lightning3.

[18] Flower DR (1998). On the properties of bit string-based measures of chemical similarity. J Chem Inform Comput Sci.

[19] Garcia-Hernandez C, Fernández A, Serratosa F (2019). Ligand-based virtual screening using graph edit distance as molecular similarity measure. J Chem Inf Model.

[20] Gaulton A, Bellis LJ, Bento AP, Chambers J, Davies M, Hersey A, Light Y, McGlinchey S, Michalovich D, Al-Lazikani B (2012). Chembl: a large-scale bioactivity database for drug discovery. Nucleic Acids Res.

[21] Gillet VJ, Willett P, Bradshaw J (2003). Similarity searching using reduced graphs. J Chem Inform Comput Sci.

[22] Gilmer J, Schoenholz SS, Riley PF, Vinyals O, Dahl GE (2017) Neural message passing for quantum chemistry. In: International conference on machine learning, PMLR, pp 1263–1272

[23] Griffen E, Leach AG, Robb GR, Warner DJ (2011). Matched molecular pairs as a medicinal chemistry tool: miniperspective. J Med Chem.

[24] Harper G, Bravi GS, Pickett SD, Hussain J, Green DVS (2004). The reduced graph descriptor in virtual screening and data-driven clustering of high-throughput screening data. J Chem Inform Comput Sci.

[25] Irwin JJ (2008). Community benchmarks for virtual screening. J Comput Aided Mol Des.

[26] Jin W, Barzilay R, Jaakkola T (2018) Junction tree variational autoencoder for molecular graph generation. In: International conference on machine learning, PMLR, pp 2323–2332

[27] Johnson MA, Maggiora GM (1990). Concepts and applications of molecular similarity.

[28] Kriege NM, Johansson FD, Morris C (2020). A survey on graph kernels. Appl Netw Sci.

[29] Kubinyi H (1998). Similarity and dissimilarity: a medicinal chemist’s view. Perspect Drug Discov Des.

[30] Landrum G (2021) Rdkit: Open-source cheminformatics software. https://github.com/rdkit

[31] Li M, Zhou J, Hu J, Fan W, Zhang Y, Gu Y, Karypis G (2021) Dgl-lifesci: An open-source toolkit for deep learning on graphs in life science. arXiv preprint arXiv:210614232 10.1021/acsomega.1c04017 PMC8529678 34693143

[32] Lima LM, Barreiro EJ (2005). Bioisosterism: a useful strategy for molecular modification and drug design. Curr Med Chem.

[33] Ma G, Ahmed NK, Willke TL, Philip SY (2021). Deep graph similarity learning: a survey. Data Min Knowl Disc.

[34] Maggiora G, Vogt M, Stumpfe D, Bajorath J (2014). Molecular similarity in medicinal chemistry: miniperspective. J Med Chem.

[35] Maggiora GM (2006). On outliers and activity cliffswhy qsar often disappoints. J Chem Inf Model.

[36] Martin YC, Kofron JL, Traphagen LM (2002). Do structurally similar molecules have similar biological activity?. J Med Chem.

[37] Méndez-Lucio O, Baillif B, Clevert DA, Rouquié D, Wichard J (2020). De novo generation of hit-like molecules from gene expression signatures using artificial intelligence. Nat Commun.

[38] Paszke A, Gross S, Massa F, Lerer A, Bradbury J, Chanan G, Killeen T, Lin Z, Gimelshein N, Antiga L, Desmaison A, Kopf A, Yang E, DeVito Z, Raison M, Tejani A, Chilamkurthy S, Steiner B, Fang L, Bai J, Chintala S, Wallach H, Larochelle H, Beygelzimer A, d’Alché Buc F, Fox E, Garnett R (2019). Pytorch: an imperative style, high-performance deep learning library. Advances in Neural Information Processing Systems 32.

[39] Patani GA, LaVoie EJ (1996). Bioisosterism: a rational approach in drug design. Chem Rev.

[40] Patterson DE, Cramer RD, Ferguson AM, Clark RD, Weinberger LE (1996). Neighborhood behavior: a useful concept for validation of “molecular diversity” descriptors. J Med Chem.

[41] Pedregosa F, Varoquaux G, Gramfort A, Michel V, Thirion B, Grisel O, Blondel M, Prettenhofer P, Weiss R, Dubourg V, Vanderplas J, Passos A, Cournapeau D, Brucher M, Perrot M, Duchesnay E (2011). Scikit-learn: machine learning in Python. J Mach Learn Res.

[42] Raymond JW, Willett P (2002). Effectiveness of graph-based and fingerprint-based similarity measures for virtual screening of 2d chemical structure databases. J Comput Aided Mol Des.

[43] Rhodes J, Boyer S, Kreulen J, Chen Y, Ordonez P (2007). Mining patents using molecular similarity search. Biocomputing.

[44] Riniker S, Landrum GA (2013). Open-source platform to benchmark fingerprints for ligand-based virtual screening. J Cheminformatics.

[45] Rogers D, Hahn M (2010). Extended-connectivity fingerprints. J Chem Inf Model.

[46] Rohrer SG, Baumann K (2009). Maximum unbiased validation (MUV) data sets for virtual screening based on pubchem bioactivity data. J Chem Inf Model.

[47] Schultz M, Joachims T (2004). Learning a distance metric from relative comparisons. Adv Neural Inf Process Syst.

[48] Sterling T, Irwin JJ (2015). Zinc 15 - ligand discovery for everyone. J Chem Inf Model.

[49] Stumpfe D, Bajorath J (2012). Exploring activity cliffs in medicinal chemistry: miniperspective. J Med Chem.

[50] Stumpfe D, Hu Y, Dimova D, Bajorath J (2014). Recent progress in understanding activity cliffs and their utility in medicinal chemistry: miniperspective. J Med Chem.

[51] Tan C, Sun F, Kong T, Zhang W, Yang C, Liu C (2018) A survey on deep transfer learning. In: International conference on artificial neural networks, Springer, pp 270–279

[52] Walters W, Stahl MT, Murcko MA (1998). Virtual screening-an overview. Drug Discov Today.

[53] Wang M, Zheng D, Ye Z, Gan Q, Li M, Song X, Zhou J, Ma C, Yu L, Gai Y, et al (2019) Deep graph library: a graph-centric, highly-performant package for graph neural networks. arXiv preprint arXiv:190901315

[54] Weiss K, Khoshgoftaar TM, Wang D (2016). A survey of transfer learning. J Big data.

[55] Willett P, Barnard JM, Downs GM (1998). Chemical similarity searching. J Chem Inf Comput Sci.

[56] Yang K, Swanson K, Jin W, Coley C, Eiden P, Gao H, Guzman-Perez A, Hopper T, Kelley B, Mathea M (2019). Analyzing learned molecular representations for property prediction. J Chem Inf Model.

